# Role of the Vitamin D Receptor (VDR) in the Pathogenesis of Osteoporosis: A Genetic, Epigenetic and Molecular Pilot Study

**DOI:** 10.3390/genes14030542

**Published:** 2023-02-21

**Authors:** Beatrice Gasperini, Virginia Veronica Visconti, Cinzia Ciccacci, Angela Falvino, Elena Gasbarra, Riccardo Iundusi, Maria Luisa Brandi, Annalisa Botta, Umberto Tarantino

**Affiliations:** 1 Department of Biomedicine and Prevention, University of Rome “Tor Vergata”, 00133 Rome, Italy; 2 Department of Clinical Sciences and Translational Medicine, University of Rome “Tor Vergata”, 00133 Rome, Italy; 3 UniCamillus-Saint Camillus International University of Health Sciences, 00131 Rome, Italy; 4 FIRMO Foundation, Via San Gallo 123, 50100 Florence, Italy

**Keywords:** osteoporosis, bone mineral density (BMD), fragility fracture risk, vitamin D receptor (VDR), *Cdx2*, *FokI*, *TaqI*, polymorphism, DNA methylation

## Abstract

The vitamin D receptor (VDR) regulates bone development and calcium homeostasis, suggesting a central role in musculoskeletal diseases such as osteoporosis (OP). Several studies have examined the contribution of *VDR* polymorphisms and epigenetic signatures in bone metabolism and OP risk, with sometimes inconclusive results. Our study aimed to explore the association between genetic variability, expression and the methylation pattern of *VDR* with the risk of OP in a cohort of Caucasian patients. Genomic DNA from 139 OP, 54 osteopenic (Ope) and 73 healthy (CTR) subjects were used for genotyping the rs731236 (*TaqI*), rs2228570 (*FokI*) and rs11568820 (*Cdx2*) polymorphisms of the *VDR* gene by an allelic discrimination assay. Quantitative real-time polymerase chain reaction (qRT-PCR) analysis of *VDR* expression levels and pyrosequencing analysis of a *VDR* promoter CpG island were carried out in a subcohort (25 OP and 25 CTR) of subjects. Data obtained showed a significantly higher OP risk for rs11568820 G/A and A/A genotypes (*p* = 0.05). qRT-PCR revealed lower *VDR* gene expression levels in the OP group compared to CTR subjects (*p* = 0.0009), also associated with both the rs11568820 A/A genotype (*p* = 0.03) and femoral fragility fractures (*p* = 0.05). No association was found between the methylation pattern of the region analyzed of the *VDR* promoter and its expression levels. Our results identify a significative association between *Cdx2* rs11568820 polymorphism and OP risk. In addition, the *VDR* transcriptomic profile suggests a putative interconnection with OP progression, providing a useful tool to stratify OP phenotype and fragility fracture risk.

## 1. Introduction

Osteoporosis (OP) is characterized by a reduction in bone mineral density (BMD) and qualitative changes in bone tissue, which predispose to an increased risk of fragility fractures [1]. Environmental, genetic and epigenetic factors appear to influence the variability of bone microarchitecture, a strong predictor of OP risk [2,3]. Alterations of bone and mineral metabolism appear to coexist in association with several pathological conditions. These bone impairments can be either a secondary complication of the pathological status, as in microvascular damage in systemic sclerosis patients, and/or a direct consequence of gene mutation, as in inherited endocrine tumors [4,5]. In this context, several factors involved in OP pathogenesis have been identified, but some of them have not yet been fully characterized, including the role of the vitamin D receptor (VDR) [6,7]. The *VDR* gene is located on chromosome 12 (12q12–q14) and consists of nine exons encoding for a protein consisting of 427 amino acids [8]. The VDR receptor belongs to the nuclear receptor family of ligand-activated transcription factors and induces genomic regulation of downstream targets involved in different biological functions, including calcium and phosphate homeostasis in bone metabolism [9,10]. The VDR mediates the actions of vitamin D establishing a heterodimer with the retinoid x receptor (RXR), which translocates into the nucleus and binds the vitamin D response element (VDRE) in the promoter regions of vitamin D target genes [11]. The expression and regulation of the *VDR* itself are influenced by multiple factors, including its promoter methylation [12]. Given the importance of the VDR in mediating the actions of vitamin D, its study represents an important goal in the understanding of musculoskeletal disorders pathogenesis, especially OP disease [13,14,15]. Several polymorphisms of the *VDR* gene which correlate with vitamin D levels have been explored due to their involvement in different phenotypes, including *FokI* (rs2228570; exon 2; C > T), *TaqI* (rs731236; exon 9; T > C) and *Cdx2* (rs11568820; promoter; G > A) [16,17,18]. *FokI* polymorphism is associated with an increased risk of OP in Asian women [19] and the TT *FokI* genotype correlates with high *VDR* expression in patients with Turner syndrome, supporting the hypothesis that *VDR* gene variants could modulate its expression pattern [7,12,16]. In addition, individual homozygotes for the C allele of the *TaqI* polymorphism showed a higher OP risk in Saudi and Caucasian populations [8,20,21]. Conflicting findings have been reported for *Cdx2* polymorphism, since it has been described as having a dual role in both protective and risk factors of OP pathogenesis. The *Cdx2* A/A genotype has been associated with increased BMD in a Belarusian population of postmenopausal women [22], and the A allele appears to positively modulate BMD in postmenopausal Japanese women [23]. However, the presence of the minor A allele of the *Cdx2* polymorphism was associated with a lower spine BMD, and the A/A genotype showed significantly lower expression of the *VDR* than the A/G and G/G genotypes [12,24]. This discrepancy could result from different ethnicity, environment, age, menopausal status and/or inadequate sample size of many studies [12,23,25]. In addition, epigenetic signatures, including DNA methylation, seem to modulate VDR gene expression and appear intensely involved in bone diseases [26,27]. In this context, higher vitamin D concentration was shown to be associated with higher *VDR* promoter methylation in rheumatoid arthritis (RA) patients [28]. Interestingly, recent data suggest a link between *Cdx2* genotype-specific *VDR* expression and differential *VDR* methylation, regardless of ethnicity [12], supporting the complex interaction between genetics, epigenetics and environment in OP pathogenesis. Among environmental factors, cigarette smoking seems to play an important role, as it correlated with the downregulation of serum vitamin D levels and inhibited the translocation of the VDR into the nucleus [29,30]. Decreased serum vitamin D levels have been also associated with acute and chronic diseases, such as systemic sclerosis (SSc) [31]. All these data suggest a key involvement of the VDR in bone metabolism, highlighting the presence of significant gaps that need further investigation to clarify the role played by this modulator. Our study is based on the hypothesis that *VDR* polymorphisms might alter mRNA expression levels and impact the metabolism of vitamin D, thus influencing the risk of developing OP. We therefore genotyped three SNPs in the *VDR* gene (*FokI*, *TaqI* and *Cdx2*) in a cohort of Italian patients, comprising OP, osteopenic (Ope) and healthy (CTR) subjects. *VDR* expression and methylation analyses were also carried out in OP and CTR subjects, to identify possible correlations of the genetic, epigenetic and transcriptomic profile of the *VDR* gene with the clinical phenotypes of OP patients.

## 2. Materials and Methods

### 2.1. Recruitment of Patients

The study was approved by the Ethical Board of the Policlinico Tor Vergata (approval reference number #17/21) and the Azienda Ospedaliero Universitaria Careggi (10419_oss; 1 March 2017); informed consent was obtained from all the participants. A total of 266 patients were enrolled and, based on clinical data, were divided into three experimental groups: 139 OP patients undergoing surgery for fragility fractures following low-energy trauma, 73 CTR subjects who underwent surgery for high-energy fractures and 54 Ope patients. All experimental procedures were conducted according to the World Medical Association Code of Ethics (Declaration of Helsinki). Individuals affected by malignancies, endocrine disorders affecting bone and mineral metabolism, autoimmune diseases, and bone disorders other than primary osteoporosis were excluded from the study, as well as those who underwent long-term therapy with drugs interfering with bone metabolism, sex hormone replacement therapy and/or anti-fracture and/or osteoanabolic therapies.

### 2.2. Clinical and Biochemical Parameters

Individuals were subdivided into OP, Ope, and CTR groups by clinical and biochemical characterization of bone quality, quantity and metabolism. Scans of the lumbar spine (L1–L4) and femur (total and neck) were performed using Lunar DXA equipment (GE Healthcare, Madison, WI, USA), according to the manufacturer’s recommendations, as reported previously [32]. In addition, 25-(OH)-VitD, PTH and calcium levels were measured from the venous blood of each patient. 25-(OH)-VitD and PTH levels were determined by chemiluminescence immunoassay (CLIA), while calcium levels were measured photometrically, according to the manufacturer’s recommendations.

### 2.3. DNA Extraction and Genotyping

The study protocol involved taking blood after an overnight fast and collecting it in tubes with anticoagulant. DNA was extracted from each subject’s peripheral blood using the FlexiGene DNA kit (Qiagen, Hilden, Germany), following the manufacturer’s instructions. Genotyping of the *Cdx2* (rs11568820), *FokI* (rs2228570) and *TaqI* (rs731236) polymorphisms of the *VDR* gene was performed using TaqMan probes (C__2880808_10, C__12060045_20, C___2404008_10) on Real-Time 7500 Fast PCR System (Applied Biosystems). A total of 20 ng of genomic DNA from each individual was amplified according to standard amplification protocol (95 °C for 10 min and 40 cycles of 92 °C for 15 s and 60 °C for 1 min). Genotyping detection analysis was performed using the Cloud Dashboard application (Thermo Fisher Scientific, Waltham, MA, USA). Ten percent of the genotypes at the rs11568820, rs2228570, and rs731236 loci were further confirmed by Sanger sequencing, using Applied Biosystems 3130xl Genetic Analyser (Thermo Fisher Scientific).

### 2.4. RNA Extraction and qRT-PCR Analysis

The total RNA from PBMCs preserved with TRIzol (Thermo Fisher Scientific, Waltham, Massachusetts, USA) was isolated. An amount of 200 µL of chloroform was added and centrifuged at 12,000× *g* for 15 min at 4 °C. Once the supernatant was removed, 500 µL of isopropanol was added and left on ice for 10 min. Additional centrifugation for 10 min at 12,000× *g* at 4 °C was performed. Once the pellet was isolated and washed in 75% ethanol, it was air-dried for 30 min. The purified RNA was dissolved in 30 μL of RNase-free water and subsequently was reverse transcribed into cDNA using High-Capacity Reverse Transcription kits (Thermo Fisher Scientific, Waltham, MA, USA). qPCR analysis was conducted using TaqMan probe (Hs01045843) (Thermo Fisher Scientific, Waltham, MA, USA) and TaqMan Universal Master Mix 2X following the manufacturer’s instructions. The relative difference in *VDR* gene expression between OP and CTR subjects was calculated using 2^−ΔΔCt^ normalized to *GAPDH* levels as the internal control.

### 2.5. Methylation Analysis

Bisulfite treatment of the DNA samples was performed with the EZ DNA Methylation-Gold kit (Zymo Research), following the manufacturer’s instructions. An amount of 400 ng of DNA was used for bisulfite conversion with the following conditions: 98 °C for 10 min, 64 °C for 2 ½ h, followed by 4 °C for up to 20 h. The converted DNA was purified and eluted in 10 μL of elution buffer. A total of 10 ng of each converted DNA was amplified with the PyroMark PCR kit (Qiagen, Hilden, Germany). Pyrosequencing assay, including PCR and sequencer primers, included Hs_VDR_03_PM (Qiagen, Hilden, Germany). Amplification conditions were 95 °C for 15 min, followed by 45 cycles of 94 °C for 30 s, 56 °C for 30 s and 72 °C for 30 s, with a final extension of 10 min at 72 °C. All products were sequenced using PyroMark Gold Q24 reagents (Qiagen, Hilden, Germany) in combination with the PyroMark Q24 platform (Qiagen, Hilden, Germany) according to the manufacturer’s instructions. The pyrogram traces generated with distinct peaks were subsequently analyzed and the methylation levels at different CpGs were calculated by the PyroMark Q24 software, version 2.0.7 (Qiagen, Germany).

### 2.6. Statistical Analysis

The Hardy–Weinberg equilibrium was verified by Pearson’s χ2 for all SNPs. The association analysis was performed according to additive, recessive, dominant and allelic models. Differences in allelic and genotypic frequencies between cases (OP or Ope) and controls were evaluated by Pearson’s χ2 test. Odds ratios (ORs) with 95% CI were calculated. To evaluate the possible correlation between the SNPs and the occurrence of fractures, a further genotype/phenotype correlation analysis was conducted. The analysis of variance (ANOVA) test was used to compare gene expression data among the different subgroups (OP vs. CTR, males vs. females, OP with femoral fracture (OP_FF) vs. OP with fractures at any sites (OP_ASF)) and among the different genotypic classes. Subsequently, a Pearson bivariate correlation analysis was used to evaluate the relationship of the *VDR* expression levels with BMD and *t*-score at any sites. Two-tailed *p* values less than 0.05 were considered statistically significant. Statistical analyses were performed by the SPSS program ver. 26 (IBM Corp., Armonk, NY, USA).

## 3. Results

### 3.1. Clinical Characteristics of Enrolled Subjects

A total of 266 subjects were enrolled and stratified into three groups after clinical diagnosis: 139 OP (116 females and 23 males), 73 CTR (41 females and 32 males) and 54 Ope (45 females and 9 males). The detailed clinical characteristics of the study subjects are shown as the mean ± SD and summarized in Table 1. Unfortunately, the group of Ope patients came to our center with only the clinical diagnosis, lacking instrumental and biochemical parameters. The mean age was 65.1 ± 7.9 in OP patients and 60.6 ± 12.1 in CTR subjects. The mean body mass index (BMI) was significantly lower in the OP group than in the CTR (*p* < 0.0001). BMD and *t*-score values at any sites observed in the OP group were significantly lower than in the CTR (*p* < 0.0001). In addition, there was no statistically significant difference in circulating markers of bone metabolism between OP and CTR groups, except for PTH (Pg/mL) levels, which were lower in OP patients compared to the CTR (52.2 ± 28.2 vs. 65.1 ± 33.3, *p* = 0. 0.0130).

### 3.2. Genotype Analysis of VDR Gene Polymorphisms

We analyzed the genotypic frequency distribution of three *VDR* polymorphisms (rs731236, *Taq1*; rs2228570, *Fok1*; rs11568820, *Cdx2*) between OP, Ope and CTR subjects. All SNPs were in Hardy–Weinberg equilibrium in all groups. Genotyping data and case-control association analysis are summarized in Table 2 (only the *p*-value related to the dominant model has been reported). The analysis showed a higher risk of developing OP (OR = 1.81, 95% CI 0.99–3.31) in carriers of the homozygous variant (A/A) or heterozygous (G/A) genotypes of the rs11568820 (*Cdx2*) polymorphism, although with a borderline significance (*p* = 0.05). No significant differences were found in the genotype’s distribution of rs731236 (*Taq1*) and rs2228570 (*Fok1*) polymorphisms between OP and CTR subjects. Furthermore, no associations have been found with the additive, recessive and allelic models for all the polymorphisms analyzed (data not shown).

### 3.3. VDR Expression Level Is Significantly Downregulated in PBMCs from OP Patients

**Figure 1:**
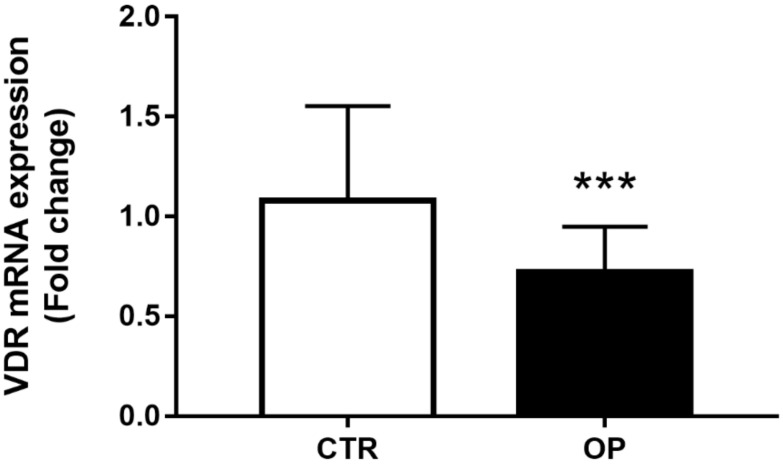
PBMC expression level of *VDR* in OP patients (n = 25) and CTR (n = 25). *GAPDH* mRNA level was used to normalize the amount of *VDR* and expression values are expressed as 2^−ΔΔCt^. Statistical differences between OP and CTR groups were analyzed by a non-parametric Mann–Whitney *U*-test (*** *p* = 0.0009).

*VDR* expression levels in PMBCs were analyzed in a subcohort consisting of 25 OP and 25 CTR individuals. Our results showed that *VDR* expression levels related to *GAPDH* were significantly lower in OP patients than in controls (Figure 1; *p* = 0.0009). Next, to investigate a possible gender-dependent effect on the level of *VDR* expression, we performed our analysis on female subjects and male subjects separately. We further confirmed the statistically significant decrease in *VDR* expression levels either in OP female or male subjects, compared to CTR, respectively (Figure S1; *p* = 0.0461 vs. *p* = 0.0012).

### 3.4. Association of VDR rs11568820 Polymorphism and VDR Expression Levels in PBMCs from OP Patients

**Figure 2:**
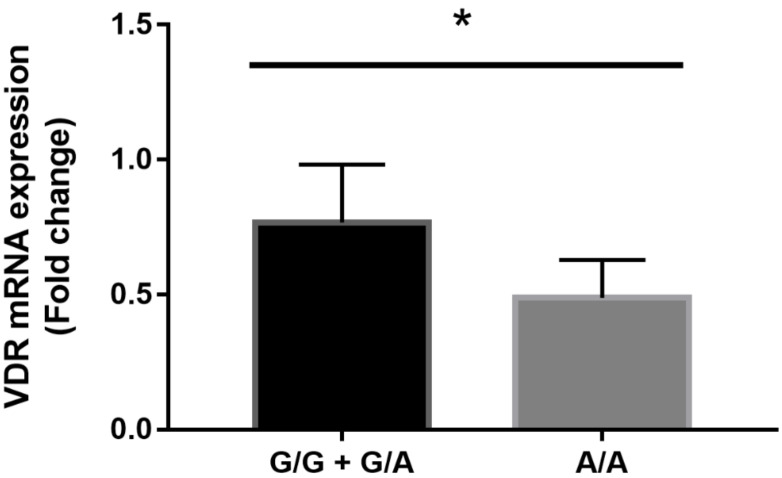
Expression level of the *VDR* gene in a subcohort composed of 25 OP patients with different genotypes of the *VDR* rs11568820 polymorphism: A/A (n = 4), G/G (n = 12) and G/A (n = 9). Statistical differences between G/G + G/A and A/A genotypes were analyzed by a non-parametric Mann–Whitney *U*-test (* *p* = 0.03).

In order to determine whether *VDR* rs2228570, rs731236 and rs11568820 polymorphisms could represent modulating factors in OP risk, we explored the effects of these *VDR* variants on the *VDR* mRNA expression pattern. Interestingly, our analysis identified a relationship between the *Cdx2* A/A genotype and *VDR*-altered expression levels in OP patients. OP patients with G/G and G/A genotypes showed higher *VDR* expression levels than those with A/A genotype (*p* = 0.03) (Figure 2). Therefore, the A/A genotype could be considered a risk factor associated with OP and fragility fractures, as a decrease in *VDR* expression levels correlates with a higher probability of developing the pathological phenotype in our pilot study cohort.

### 3.5. VDR Expression Levels Correlate with Bone Fragility Status

**Figure 3:**
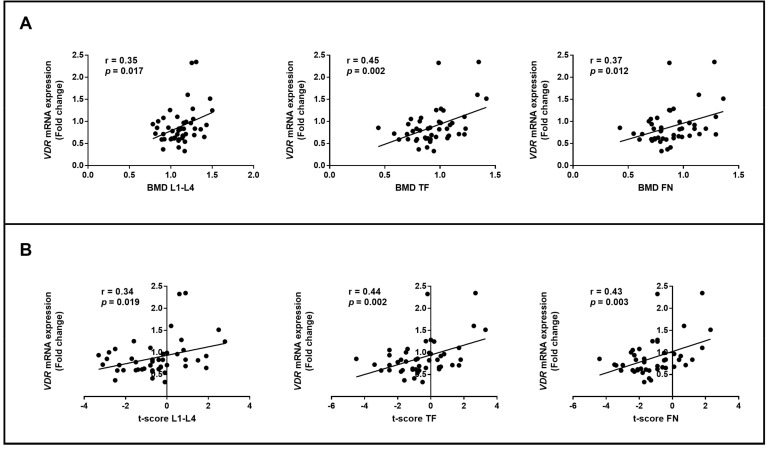
Correlation between *VDR* expression levels and instrumental clinical characteristics of OP and CTR subjects. *VDR* expression levels were determined by qRT-PCR; each point represents one subject. *VDR* levels were positively associated with BMD (**A**) and *t*-score (**B**) values of the L1–L4 lumbar spine, total femur and femoral neck. Pearson’s correlation values (r) are indicated in each graph.

Differences between *VDR* mRNA expression levels and clinical instrumental characteristics of OP patients and healthy controls were assessed using Pearson correlation analysis. This analysis showed a significant positively correlation between *VDR* expression levels and both BMD and *t*-score values, at the lumbar spine, total femur and femoral neck, within all enrolled subjects (Figure 3).

Furthermore, to investigate a possible relationship between *VDR* expression level and fragility fracture site, OP patients were divided into two groups consisting of 15 patients with femoral fractures (OP_FF) and 10 patients with fractures at any sites (OP_ASF). Our analysis identified a significant difference in *VDR* expression levels between OP_FF and OP_ASF (*p* = 0.05) (Table 3). OP_FF patients reported lower mean *VDR* expression levels, which could be associated with a more severe pathological phenotype, in which the index fracture is that of the femur.

### 3.6. Methylation Levels of the CpG Island in the Promoter Region of the VDR Gene

**Figure 4:**
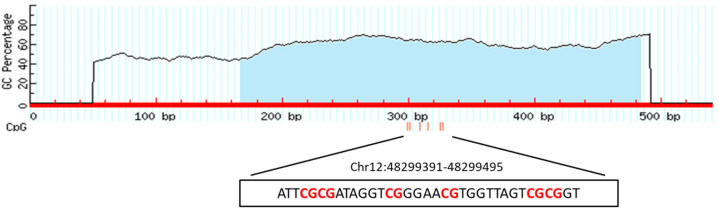
Methylation analysis of promoter CpG island located in *VDR* locus. CpG island (blue region) identified by MethPrimer program and location of the CpG sites in *VDR* gene.

In order to investigate the contribution of epigenetic signatures modulating *VDR* expression in OP pathogenesis, we analyzed the methylation status of a CpG located in the promoter region both in OP (n = 25) and CTR (n = 25) subjects. The methylation analysis was performed in a 104 bp region, comprising six CG sites located in chr12:48299391-48299495 (Figure 4).

Unfortunately, our pyrosequencing analysis showed no statistically significant difference in the methylation pattern of the *VDR* gene between OP and CTR groups, as we found an average global methylation level of 5.3–5.1% in PBMCs of both categories (Table 4, Figure S2). Overall, we identified a slight increase in methylation levels at the CpG 3 site (6.8–7.0%) and a slight decrease at the CpG 4 site (2.8–2.9%) in both OPs and CTRs, in respect to the average methylation pattern of the other analyzed sites.

## 4. Discussion

In this study, we investigated the role of three selected polymorphisms (*TaqI*, *FokI* and *Cdx2*) of the *VDR* gene in an Italian cohort of 139 OP, 54 Ope and 73 CTR subjects. Our analysis did not reveal any significant associations between *FokI* and *TaqI* polymorphisms and OP risk. These results are in agreement with a meta-analysis including 3349 postmenopausal OP women and 3202 healthy subjects that showed no significant associations between *FokI* polymorphism and OP risk in the Caucasian population compared to the Asian population [19]. Accordingly, another meta-analysis including 31 studies showed no significant association between *TaqI*, *ApaI* and *FokI* polymorphisms and OP risk in females [33]. Interestingly, we estimated a 1.8 times more significant OP risk in carriers of the homozygous (A/A) or heterozygous (G/A) genotype of the *Cdx2* rs11568820 polymorphism. However, the role of this genetic variant in OP susceptibility is highly controversial. *Cdx2* polymorphism consists of a G > A substitution located in the *VDR* exon 1 promoter region which has been shown to alter intestinal *VDR* gene transcriptional activity [34]. A functional study based on luciferase assay, showed that the A allele of the *Cdx2* rs11568820 induces 15% increased transcriptional activity of the *VDR* gene [23]. In contrast to these findings, our data indicated a significant reduction in *VDR* expression levels in A/A genotype carriers, identifying the A allele as a risk factor for OP susceptibility, also in accordance with the genotypic analysis. In support of our results, a study performed on monocyte/macrophages from healthy Black and White individuals identified a significant association between the A/A genotype and low 25(OH) D3 levels, which are also associated with *VDR* reduced expression levels [12]. Moreover, the G/G genotype has been correlated with a better bone response to mechanical stress and OP risk [35]. These findings provide additional information regarding the contribution of the *Cdx2* rs11568820 polymorphism in bone metabolism imbalances. Indeed, it is now certain that *VDR* expression is not only dependent on the individual’s vitamin D status but also on other factors, including genetic variability [36,37]. Our pilot study in a subcohort of 25 OP and 25 CTR subjects showed that *VDR* expression levels in PMBCs were significantly decreased in OP patients, despite the two categories showing no statistically significant differences in circulating 25-(OH)-Vit D levels. In this paper, we identified for the first time a strong interconnection between *VDR* expression level and instrumental parameters of bone quality. Interestingly, we found a significant positive correlation between *VDR* mRNA expression levels and both BMD and *t*-score values at the lumbar spine, total femur and femoral neck loci in all subjects. BMD assessment using the DXA technique represents the gold standard for OP diagnosis, according to the World Health Organization (WHO) [38]. Our results showed that when *VDR* expression levels are downregulated, BMD and *t*-score decreased proportionally, associating the altered *VDR* expression pattern with a gradual stratification of bone densitometry, from the healthy to severe OP phenotype. A strength of our analysis is undoubtedly the presence of patients with severe OP, defined by the coexistence of the disease together with fragility fractures, particularly at the femur site [39]. Confirming the negative role played by the *VDR* decrease in OP pathogenesis and fracture risk, our results showed lower mean *VDR* expression levels in patients with femur fractures (OP_FF), which is considered the index fracture of OP disease. Finally, we also correlated the methylation status of a CpG island located in the *VDR* promoter region with the *VDR* gene expression levels with no significant results. Several studies have highlighted the role of methylation in modulating *VDR* expression in different pathological contexts, mainly in autoimmune diseases, but none of these has concerned OP [39]. We focused our methylation analysis in a small region including six CG sites located in a *VDR* promoter CpG island (CGI 1062) already shown to functionally modulate *VDR* expression in a South African population [12]. In this study, methylation was quantified across 56 CpG sites, whereas our pyrosequencing analysis relied on a predesigned assay including only 6 CpG sites partially overlapping with the *VDR* region analyzed by Meyer et al. The lack of correlation between *VDR* expression levels and the methylation of the CpG island analyzed by us could be attributable either to the presence of additional functionally active CpG islands/CG sites or to other modulatory mechanisms.

This is a pilot study with some limitations: firstly, the small sample size of patients and controls. On the other hand, the strength of the sampling is in the very accurate instrumental and biochemical characterization of OP and CTR individuals. Unfortunately, characterization of the Ope group is partially missing to conduct more complete correlation analyses. In addition, our cohort included more women (n = 202) than men (n = 64), but we performed sex-stratified analyses that confirmed the statistical significance of the tests used for the overall study population. Moreover, the average age of the CTR group is slightly lower than that of the OP. However, this difference is not easy to bridge due to the difficulty of recruiting individuals of the same age who are not affected by age-related bone diseases or other comorbidities. Finally, unfortunately, the subcohort on which we performed the *VDR* expression analysis is limited to only 50 subjects, including 25 OP and 25 CTR participants, whose PBMCs were available for RNA extraction.

## 5. Conclusions

The contribution of *VDR* genetic variability in OP pathogenesis is still unclear and full of gaps. The fluctuation in the literature data can be attributed to several aspects, including the different allele distribution between ethnicities, which is crucial in defining disease susceptibility. This study enlarges our knowledge on the role of *VDR* genetic variability in OP susceptibility in the Italian population. In addition, the transcriptomic profile of the *VDR* gene suggests a putative key interconnection with OP progression, providing a useful tool for stratifying OP phenotype and fracture risk. The *VDR* circulating altered expression pattern could also candidate this factor as a potential non-invasive biomarker for the early diagnosis of age-related OP disease. Notably, a novel biomarker could significantly improve the quality of life of the elderly population, also reducing individual and hospital care costs, factors that actually make OP a major burden worldwide.

## Figures and Tables

**Table 1:** Clinical features of OP, CTR and Ope subjects.

	OP (n = 139)	CTR (n = 73)	Ope (n = 54)	*p* Value
Age (years)	65.1 ± 7.9	60.6 ± 12.1	na	* (*p* = 0.0167)
BMI (kg/m^2^)	24.1 ± 3.8	27.6 ± 5.2	na	**** (*p* < 0.0001)
BMD L1–L4	0.8 ± 0.2	1.2 ± 0.1	na	**** (*p* < 0.0001)
*t*-score L1–L4	−2.4 ± 1.3	0.3 ± 1.0	na	**** (*p* < 0.0001)
BMD TF	0.7 ± 0.1	1.0 ± 0.1	na	**** (*p* < 0.0001)
*t*-score TF	−2.0 ± 1.0	0.4 ± 1.0	na	**** (*p* < 0.0001)
BMD FN	0.6 ± 0.1	0.9 ± 0.1	na	**** (*p* < 0.0001)
*t*-score FN	−2.4 ± 0.8	−0.2 ± 0.9	na	**** (*p* < 0.0001)
Calciulm (mg/dL)	8.9 ± 0.9	8.9 ± 0.6	na	NS (*p* = 0.3600)
PTH (Pg/mL)	52.2 ± 28.2	65.1 ± 33.3	na	* (*p* = 0.0130)
25-(OH)-Vit D (ng/mL)	24.4 ± 11.9	24.2 ± 9.1	na	NS (*p* = 0.8108)

BMI, body mass index; BMD, bone mineral density; FN, femoral neck; TF, total femur; PTH, parathyroid hormone; 25-(OH)-Vit D, 25-hydroxyvitamin D; * *p* < 0.05, **** *p* < 0.0001.

**Table 2:** Genotype distribution of *VDR* polymorphisms.

rs731236 A > G *Taq1*	TOT	T/T	T/C	C/C	*p* ^a^	OR (95% CI)
OP	139	41	69	29	0.48	1.25 (0.68–2.28)
OPE	54	16	26	12	0.58	1.24 (0.58–2.64)
CTR	73	25	34	14		
rs2228570 C > T *Fok1*	TOT	C/C	C/T	T/T	*p* ^a^	OR (95% CI)
OP	135	54	62	23	0.54	1.25 (0.68–2.28)
OPE	54	17	29	8	0.8	1.24 (0.58–2.64)
CTR	73	25	34	14		
rs11568820 G > A *Cdx2*	TOT	G/G	G/A	A/A	*p* ^a^	OR (95% CI)
OP	139	78	52	9	*0.05*	1.81 (0.99–3.31)
OPE	54	35	18	1	0.55	1.26 (0.6–2.66)
CTR	73	51	19	3		

*p*^a^ value represents the analysis based on the dominant model (heterozygous and homozygous variant genotypes were grouped in a single class)). OR: odds ratio; CI: confidence interval. Significant associations are reported in italics.

**Table 3:** Association between *VDR* mRNA expression levels and the fracture bone site of the OP group.

	*VDR mRNA* Expression (Fold Change Mean ± SD)	*p*
OP_FF	0.65 ± 0.19	0.05
OP_ASF	0.83 ± 0.25	

**Table 4:** Methylation analysis of *VDR* promoter CpG island.

Mean CpG Sites Meth%	OP (n = 25)	CTR (n = 25)
CpG 1	5.4	5.5
CpG 2	5.1	5.0
CpG 3	7.0	6.8
CpG 4	2.9	2.8
CpG 5	6.4	5.9
CpG 6	4.8	4.8
Mean Global Meth%	5.3	5.1

## Data Availability

The data presented in this study are available on request from the corresponding author.

## Supplementary Materials

The following supporting information can be downloaded at https://www.mdpi.com/article/10.3390/genes14030542/s1, Figure S1: PBMCs expression level of VDR in OP female and male patients, Figure S2: Pyrosequencing profiles of a representative OP patient.
