# Supplementary material for: Role of the Vitamin D Receptor (VDR) in the Pathogenesis of Osteoporosis: A Genetic, Epigenetic and Molecular Pilot Study
Source: Genes (Basel). 2023 Feb 21;14(3):542. doi: 10.3390/genes14030542 (PMC10048253; doi:10.3390/genes14030542)
Supplement: Supplementary file 1 [file genes-2221875-supplementary.pdf › genes-2221875-supplementary.pdf]

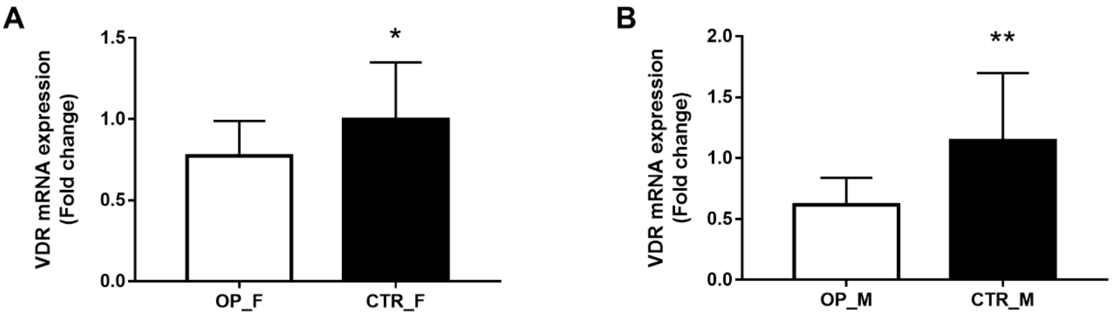

Figure S1. PBMCs expression level of *VDR* in OP female and male patients. (**A**) *VDR* expression level was analyzed in the PBMCs of 17 OP total female and 11 healthy female controls. * $p < 0.0461$; (**B**) *VDR* expression level was analyzed in the PBMCs of 8 OP total male and 14 healthy male controls. * $p < 0.0012$. *GAPDH* mRNA level was used to normalize the amount of *VDR* and expression values are expressed as $2^{-\Delta\Delta Ct}$.

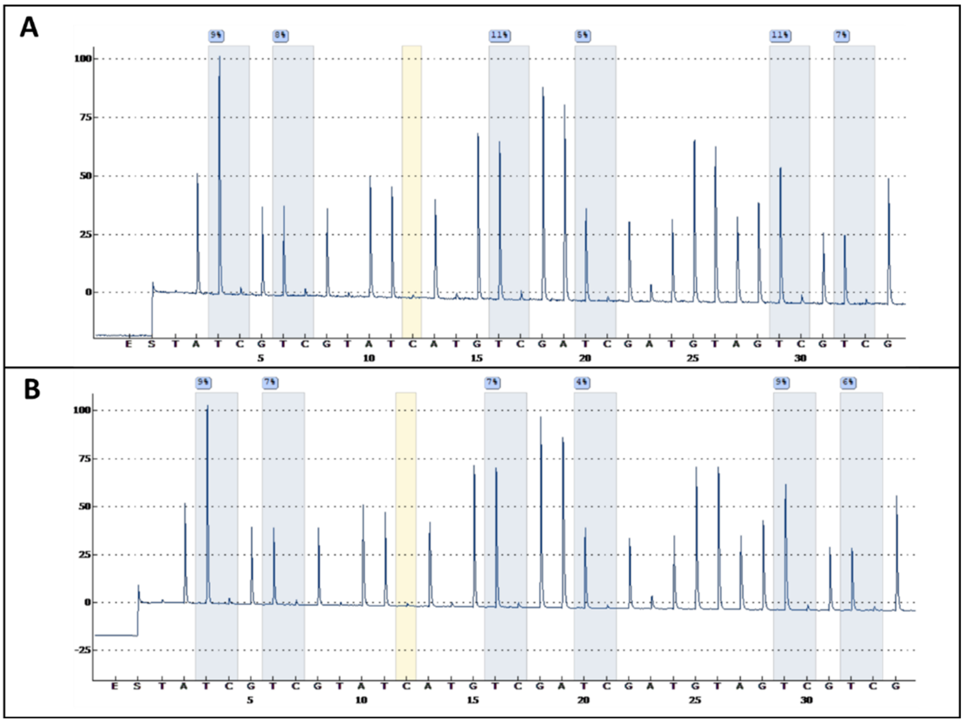

Figure S2. Pyrosequencing profiles of a representative OP patient (up-panel) and a healthy subject (lower panel) analysed in this study.
